# Computational Insights into Acrylamide Fragment Inhibition of SARS-CoV-2 Main Protease

**DOI:** 10.3390/cimb46110765

**Published:** 2024-11-12

**Authors:** Ping Chen, Liyuan Wu, Bo Qin, Haodong Yao, Deting Xu, Sheng Cui, Lina Zhao

**Affiliations:** 1 CAS Key Laboratory for Biomedical Effects of Nanomaterials and Nanosafety, Institute of High Energy Physics, Chinese Academy of Sciences, Beijing 100049, China; chenping@ihep.ac.cn (P.C.); wuly@ihep.ac.cn (L.W.); yaohd@ihep.ac.cn (H.Y.); xudt@ihep.ac.cn (D.X.); 2 University of Chinese Academy of Sciences, Beijing 100049, China; 3 NHC Key Laboratory of Systems Biology of Pathogens, Institute of Pathogen Biology, Chinese Academy of Medical Sciences & Peking Union Medical College, Beijing 100730, China; qinbo@pumc.edu.cn (B.Q.); cui.sheng@ipb.pumc.edu.cn (S.C.); 4 Key Laboratory of Pathogen Infection Prevention and Control (Peking Union Medical College), Ministry of Education, Beijing 100730, China

**Keywords:** SARS-CoV-2 Mpro, acrylamide fragments, molecular dynamics simulation, novel antiviral drugs, molecular mechanism

## Abstract

The pathogen of COVID-19, SARS-CoV-2, has caused a severe global health crisis. So far, while COVID-19 has been suppressed, the continuous evolution of SARS-CoV-2 variants has reduced the effectiveness of vaccines such as mRNA-1273 and drugs such as Remdesivir. To uphold the effectiveness of vaccines and drugs prior to potential coronavirus outbreaks, it is necessary to explore the underlying mechanisms between biomolecules and nanodrugs. The experimental study reported that acrylamide fragments covalently attached to Cys145, the main protease enzyme (Mpro) of SARS-CoV-2, and occupied the substrate binding pocket, thereby disrupting protease dimerization. However, the potential mechanism linking them is unclear. The purpose of this work is to complement and validate experimental results, as well as to facilitate the study of novel antiviral drugs. Based on our experimental studies, we identified two acrylamide fragments and constructed corresponding protein-ligand complex models. Subsequently, we performed molecular dynamics (MD) simulations to unveil the crucial interaction mechanisms between these nanodrugs and SARS-CoV-2 Mpro. This approach allowed the capture of various binding conformations of the fragments on both monomeric and dimeric Mpro, revealing significant conformational dissociation between the catalytic and helix domains, which indicates the presence of allosteric targets. Notably, Compound **5** destabilizes Mpro dimerization and acts as an effective inhibitor by specifically targeting the active site, resulting in enhanced inhibitory effects. Consequently, these fragments can modulate Mpro’s conformational equilibrium among extended monomeric, compact, and dimeric forms, shedding light on the potential of these small molecules as novel inhibitors against coronaviruses. Overall, this research contributes to a broader understanding of drug development and fragment-based approaches in antiviral covalent therapeutics.

## 1. Introduction

The coronavirus family has given rise to three significant epidemics: Severe Acute Respiratory Syndrome (SARS), Middle East Respiratory Syndrome (MERS), and the ongoing Coronavirus Disease 2019 (COVID-19) pandemic [1]. COVID-19 has caused a global health crisis, characterized by an unprecedented rate of mortality, infection, and transmission, particularly affecting unvaccinated elderly individuals [2]. Symptoms range from mild, such as fever, cough, and shortness of breath, to severe cases that lead to pneumonia and even death [3]. SARS-CoV-2, the causative agent of COVID-19, shares a high degree of genetic homology with SARS-CoV, demonstrating alarming transmissibility, a heightened risk of reinfection, and a reduction in vaccine efficacy against emerging variants. SARS-CoV-2, a single-stranded, crown-shaped, positive sense RNA virus, expresses two polyproteins, pp1a and pp1ab, encoded by genomic RNA and subsequently cleaved by main protease (Mpro) or papain-like protease (PLpro) [4,5]. Recent research has highlighted the ongoing evolution of SARS-CoV-2, with new variants such as Omicron leading to changes in transmission dynamics and reduced drugs or vaccine effectiveness [6,7,8]. These developments emphasize the need for updated therapeutic approaches and adaptive vaccine strategies to address these evolving threats [9,10]. The continuous evolution of SARS-CoV-2 mutants reduced the efficacy of nucleoside analog, GS-5734 (remdesivir), MK-4482 (known previously as EIDD-2801) [11], and vaccines for Moderna (mRNA-1273) and Pfizer-BioNTech (BNT162b2) [12], thereby causing intense attention to develop effective antiviral drugs and vaccines to combat COVID-19. However, traditional vaccine development and de novo drug design processes are both time-consuming and costly. Besides, the clinical availability of new drugs remains uncertain. In the face of the growing impact of COVID-19, the concept of drug repurposing, encompassing strategies such as repositioning, re-profiling, or rediscovery, emerges as a pragmatic approach to identifying potential therapeutics [13]. Within the realm of drug discovery, Compounds can be categorized as covalent or non-covalent, with the former presenting several advantages, including enhanced biochemical efficiency, target specificity, safety, and reduced drug resistance [14]. Consequently, most studies have concentrated on covalent drug discovery.

In the quest for effective antiviral drugs, structural and biochemical research on SARS-CoV-2 represents a promising avenue, offering insights into its mechanisms and means of regulating protein activity, thereby accelerating antiviral drug development. Research shows that Mpro from coronavirus functions as a symmetric homodimer. This homodimer consists of two protomers, designated as chains A and B, with each protomer consisting of three domains (I, II, and III) connected by long-loop regions [15,16]. Domains I and II belong to the barrel fold and are located in the catalytic site of Mpro, and domain III comprises an α-helical domain. Mpro plays a pivotal role in cleaving precursor polyproteins into individual, functional, mature, non-structural polypeptides responsible for the production of critical structural proteins, including the nucleocapsid protein (N), membrane glycoprotein (M), small envelope glycoprotein (E), and spike protein (S) [17]. The spike protein serves as the entry point for SARS-CoV-2 into host cells, mediating binding with angiotensin-converting enzyme-2 (ACE2) and initiating the infection process [18]. Notably, previous studies indicate that only the dimeric form is active in the biological medium, highlighting the importance of Mpro dimerization in its function [19]. Particularly, Mpro stands as an ideal drug target for the treatment of COVID-19 and the control of SARS-CoV-2 infection.

As a complement and validation to the experimental report, we have constructed molecular models of two covalent fragment structures (Compounds **2** and **5**) in Table 1. Among seven fragments, these two fragments have exceptional inhibitory potency [20]. Compounds **2** and **5** were selected for detailed study based on experimental analyses. In vitro assays demonstrated that these two Compounds exhibited the lowest half-maximal inhibitory concentrations (IC50 = 10–20 µM) among the acrylamide fragments tested. Additionally, size-exclusion chromatography (SEC) was performed to analyze the molecular weight distribution of the Mpro-ligand complexes. The results showed that Compound **2** eluted in a dimeric form, while Compound **5** eluted predominantly as a monomer. This suggests that Compound **5** may induce a more significant disruption of Mpro dimerization, a key factor in its activity. Therefore, these two fragments were selected for further computational study. The covalent fragments share a common thiazole motif, which underpins their binding mechanism. The thiazole rings with sulfur atoms can interact with the active site cysteine (Cys145) in Mpro dimers or monomers. Despite their shared thiazole core and methylene linker, these fragments exhibit both similarities and differences. In terms of similarity, both fragments feature cysteine-reactive chemical groups that functionalize their core scaffolds, conferring the same 1,3-thiazole core and methylene linker. Alkyl acrylamide displays low off-target reactivity and limited reactivity with glutathione, while trifluoromethyl and cyclohexane fragments possess weaker electron density, minimizing interactions with the protein. Diverging from these similarities, Fragment 5, with its bulky benzene moiety, expands the substrate binding pocket, influencing neighboring regions. Especially in contrast to trifluoromethyl fragments, the benzene ring on Fragment 5 is able to form π-π stacks interaction with residue His of the target protein, thereby enhancing the stability of the protein-ligand complex. These variations in pharmacophores account for the differing inhibitory effects of these fragments on enzyme activity. Electrophiles on these fragments maintain a high level of pharmacological safety, owing to their widespread clinical application [21]. Furthermore, their intrinsic reactivity promotes protein modification and reduces false positives and negatives. The acrylamide “warhead” component is a vital feature in clinically approved covalent drugs. Emphasizing the importance of selectivity and reactivity, as well as mild electrophilic reactivity, it can collectively minimize the risk of non-specific reactivity and associated toxicity [22,23]. Collectively, acrylamide fragments offer the potential to influence intermediate states of SARS-CoV-2 Mpro by covalently targeting the active site cysteine, also providing valuable insights for rational fragment-based drug design and discovery against coronaviruses.

In this study, we explore the underlying mechanisms of two acrylamide fragments targeting Mpro through computational chemistry methods. We aim to provide important theoretical support and a molecular-level understanding of the inhibition mechanism by acrylamide fragments, which can accelerate the development of novel drugs based on acrylamide scaffolds. For example, Yu et al. designed and synthesized a series of novel 5-cyano-2,4,6-substituted pyrimidine derivatives based on acrylamide fragments. They explored the effects of the electronic properties and substitution positions of different Compounds on antitumor activity [24], demonstrating that our findings could similarly guide the rational design of new small-molecule drugs targeting viral proteases, building upon fragment-based approaches. We analyzed their ability to disrupt Mpro dimerization and trapped their transient conformation among extended, compact, and active states. Further analysis of simulated data confirmed that these two acrylamide fragments align with previous studies, confirming the utility of acrylamide moieties in several clinically approved drugs. It is worth noting that there are already existing inhibitors and early fragment-based drug design studies in this domain, such as penicillin, omeprazole (a proton pump inhibitor), and acetylsalicylic acid (aspirin) [25,26]. Early fragment-based drug design studies have laid a foundation for our research, providing crucial insights into the feasibility of targeting viral proteases [27]. These studies serve as vital precedents, guiding our approach toward the development of potential COVID-19 therapeutics. Therefore, this work is helpful in designing potential anti-coronavirus drugs.

## 2. Materials and Methods

Preparation of Mpro and Ligands: The three-dimensional structure of SARS-CoV-2 Mpro utilized in the simulations was obtained from PDB code 7WYP, with a crystal resolution of 2.30 Å (https://www.rcsb.org/3d-view/7WYP, accessed on 16 February 2022). Additionally, an apo-enzyme structure (unliganded SARS-CoV-2 Mpro dimer) was used to explore how Mpro dimerization is disrupted by modification with Compound **5** [20]. The Mpro dimer consists of two chains, A and B, with each chain representing a monomeric Mpro containing 300 and 305 residues, respectively. For the monomeric system, chain B was used for simulation. Each Mpro structure was optimized using the amber99sb-ildn force field in GROMACS 2021.3 [28] to obtain the most stable conformation as the starting structure. For the ligands, the structure files of the acrylamide fragments were first generated using GaussView 6.0 software (Version 6.0, Roy Dennington, Todd A. Keith, and John M. Millam, Semichem Inc., Shawnee Mission, KS, USA, 2016). Subsequently, geometry optimization was performed using Gaussian 16 [29], with the functional basis set B3LYP/6-31G*.

Construction of Initial Protein-Ligand Complex: AutoDock 1.5.7 was first employed to conduct molecular docking studies, confirming the binding interaction of the alkene carbon of acrylamide with the catalytic cysteine residue Cys145, which is a well-defined binding site. Subsequently, Packmol was utilized to generate the initial configuration for molecular dynamics simulations of the Mpro-antiviral drug complex [30].

Molecular Dynamics Simulations: Molecular dynamics (MD) simulations were conducted using GROMACS 2021.3 to study the inhibition mechanism between the protein and ligand. The topology for the enzyme was generated using the GROMACS command pdb2gmx, employing the amber99sb-ildn force field, which handles standard amino acids within the protein without requiring additional modifications. Also, the TIP3P water model was used for the protein topologies. For the ligand (acrylamide fragments), Sobtop 1.0 (Tian Lu, Sobtop 1.0 (dev5), http://sobereva.com/soft/Sobtop (accessed on 15 September 2024)) was employed to generate the force field parameters. While GAFF defines bond and angle parameters, these may differ from those derived via high-quality Hessian matrices, particularly for unusual bonding patterns. When generating GROMACS topology files with Sobtop, unrecognized GAFF atom types are assigned UFF types, with missing parameters calculated from the Hessian, potentially reducing the accuracy of force field parameters. The final topology files do not contain atomic charges, which are computed separately using the RESP method in Multiwfn [31]. For more accurate topologies, potential energy surface (PES) scans of the dihedral angles could be performed to obtain refined parameters, although this is time-consuming. Importantly, building the topology for the ligand and the enzyme involved constructing a molecular model with a covalent bond between the alkene carbon of acrylamide and the sulfur of Cys145, followed by optimization in Gaussian and calculating atomic charges using Multiwfn. Cys145 was defined as a new CYU residue (deprotonated Cys145) in amber99sb-ildn/aminoacids.rtp, with updated atomic charges. The topology for Mpro was then generated using the pdb2gmx command, ensuring Cys145 was in its deprotonated state.

In the MD simulation, the pH was set to a neutral state (pH 7), consistent with physiological conditions, to accurately model protein-ligand interactions. Specifically, all ionizable acidic residues, such as Asp and Glu, were modeled in their deprotonated forms. Basic residues, including Lys, Arg, and His, were maintained in their protonated states, with His residues represented as HIE. Regarding Cys145, it was treated in its deprotonated form to realistically represent its covalent bond with the ligand’s acrylamide moiety, aligning with the experimental conditions.

Each system was independently run, and energy minimization was performed using the steepest descent algorithm for 1000 steps to ensure a well-relaxed starting structure. The temperature was maintained at 300 K, and the pressure was set at 1 atm. Van der Waals (VDW) interactions were treated using a switch function with a cutoff distance of 1.4 nm. The Particle Mesh Ewald (PME) method was applied to handle long-range electrostatics with a cutoff of 1.2 nm and a Fourier grid spacing of 1.2 nm. Before performing production MD simulations, each system underwent NVT and NPT simulations to reach a pre-equilibrated state. The NVT ensemble was simulated for 5 ns using the v-rescale algorithm and Berendsen thermostat. The NPT ensemble for 5 ns was carried out with positional restraints on the complexes. The restraints on the complexes were removed in normal MD simulation for 300 ns. Where computational resources allowed, longer pre-equilibration simulations and production MD runs were also considered. All simulation snapshots were visualized using the VMD program [32].

Analyses of Structural Dynamics: Principal Component Analysis (PCA) was conducted using GROMACS 2021.3 software, specifically employing the “gmx covar” and “gmx anaeig” modules. Before PCA was performed, the trajectory underwent periodic correction to eliminate any periodic boundary effects, and translational and rotational motions were removed using the -fit rot+trans option. The PCA process involved reducing the 3N Cartesian coordinates of the molecular dynamics simulation trajectory to construct a covariance matrix, followed by the calculation of its eigenvalues and eigenvectors to describe the main molecular motions, referred to as the principal components. The two largest eigenvectors were selected as the primary components (PC1 and PC2). Additionally, the parameters describing the conformational free energy changes—RMSD and radius of gyration—were projected onto the first two principal components to capture the characteristics of the conformational changes. Besides, the molecular mechanics Poisson-Boltzmann Surface Area (MM-PBSA) [33] method was employed to calculate the relative binding free energies of the acrylamide fragments to Mpro. The binding free energy is described as ΔG_bind_ = ΔH − TΔS, where ΔH = Δ_Eelec_ + ΔE_vdW_ + ΔG_polar_ + ΔG_non-polar_. Here, E_elec_ and E_vdW_ represent the electrostatic and van der Waals energies, while G_polar_ and G_non-polar_ represent the solvation energies. The final 10 ns of the equilibrated trajectories were selected for the MM-PBSA calculations. MM-PBSA calculations were based on 10 ns of equilibrated trajectories with periodic boundary corrections, and the final binding free energy analysis was performed using the gmx_MMPBSA_ana tool.

The analyses of root mean square deviation (RMSD), root mean square fluctuation (RMSF), and radius of gyration were performed using GROMACS. The RMSD was calculated based on the entire protein backbone to capture the overall structural dynamics and conformational changes throughout the simulation. The RMSF analysis utilized the complete dynamics trajectory, enabling an assessment of fluctuations for each atom or residue over the entire simulation period, thereby providing a comprehensive view of the protein’s dynamic behavior and flexibility. For the radius of gyration, the analysis also included the entire protein, allowing for an accurate evaluation of overall compactness and structural dynamics throughout the simulation.

Various software tools were used for visualization and comprehensive analysis, including VMD 1.9.3 and PyMOL 4.5.0 (https://en.wikipedia.org/wiki/PyMOL, accessed on 12 March 2024) for molecular visualization. Python 3.7.9 scripts were employed for custom data processing and analysis. Additionally, Origin 2024 and Excel were used for graphical representation and statistical analysis of the results. To further minimize randomness and errors, we conducted two additional independent 300ns MD simulations, and the analyses demonstrated consistency in the conclusions drawn from all three MD simulations. The relevant molecular data have been incorporated into the Supplementary Materials (Supplementary Tables S1–S3).

## 3. Results and Discussion

### 3.1. Exploring the Transient States of Dimeric and Monomeric SARS-CoV-2 Mpro upon Binding with Different Acrylamide Fragments

**Figure 1:**
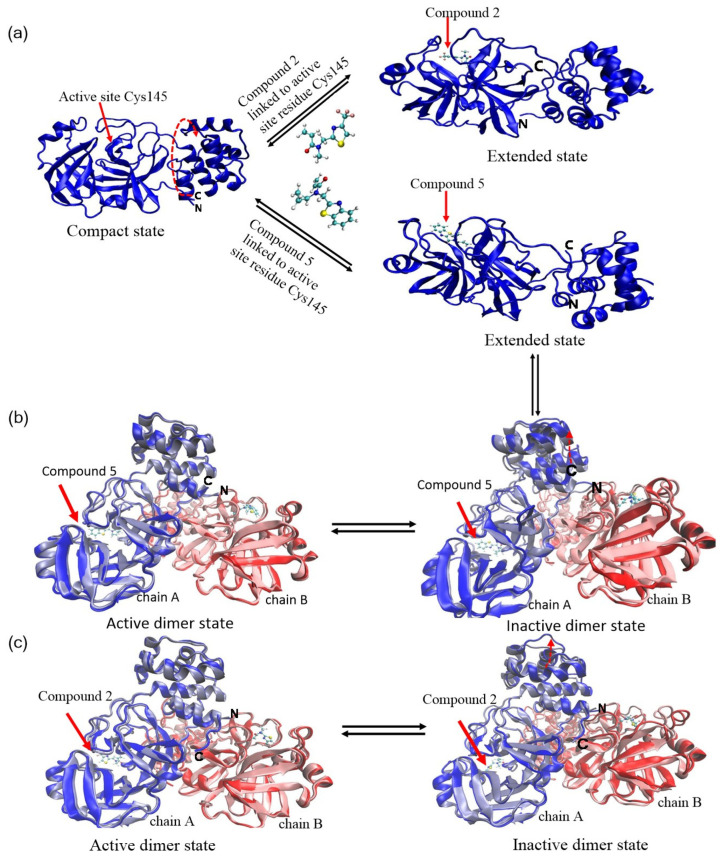
(**a**) Transient structural conformation of SARS-CoV-2 monomeric Mpro bound to Compounds 2 and 5. Transient conformation of SARS-CoV-2 dimeric Mpro modified by (**b**) Compound **5** and (**c**) Compound **2**. Superposition of the compact state, active protomer of the dimer, and the extended Mpro, colored navy blue and red. Light blue and pink represent the active dimeric Mpro without Compounds. Red arrow indicates the position of the small molecule at the active site.

**Figure 2:**
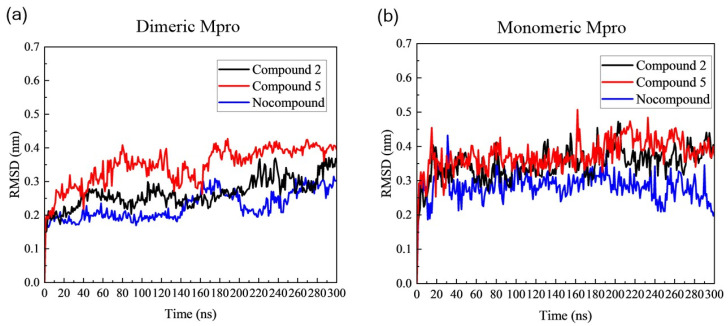
This RMSD of (**a**) dimeric Mpro and (**b**) monomeric Mpro in the apo form, both without Compounds and covalently attached to Compounds 2 and 5.

To elucidate the inhibitory mechanisms and binding affinity of acrylamide fragments (Compound **5** and Compound **2**) on SARS-CoV-2 Mpro, we captured the transient structures of dimeric and monomeric Mpro covalently bound to Compound **5** and Compound 2 (Figure 1). Crystallography of the monomeric Mpro linked to Compound **5** and Compound **2** reveals their binding models to the catalytic domain (barrel fold) and substrate binding site. Compound **2** and Compound **5** covalently attach to the active site cysteine Cys145, situated within the cleft between domain I and domain II of the barrel fold. The loop-loop interaction is responsible for the contact between the C-terminal and N-terminal. An abundance of literature revealed that the C- and N-terminal domains play pivotal roles in the maturation, dimerization, and enzyme activity of Mpro [34,35,36]. It is observed that Compound **2** bound to Mpro caused slighter displacement and rotation of the C-terminal and N-terminal, while Mpro with Compound **5** showed an obvious upward rotation of about 180° and twisting motion at the C-terminal domain (Figure 1a). Notable conformational changes occurred at 180 ns (Figure 2), leading to global backbone alterations that resulted in the disruption of the α-helical domain and the antiparallel barrel fold, further destroying the secondary structure of the protein. The root mean square fluctuation (RMSF) value of monomeric Mpro bound to Compound **5** is larger than that bound to Compound **2**, with average values of 0.174 nm and 0.159 nm, respectively. In particular, residues 90–100, 218–222, and 300–305 fluctuate widely (Figure S1b), suggesting unstable configurations. By contrast, Compound **2** linked to monomeric Mpro and the C-terminal finger of the -helical domain exhibited about 30 swings relative to the monomeric Mpro without ligands, which partly released the constraints of the C-terminal of Mpro. It is worth noting that the binding of the acrylamide fragments did not induce a fully conformational change to the independent fold units (-helical or -barrel). Notably, Mpro with Compound **5** caused striking structure fluctuation and destruction. Hence, Compound **5** may be a potential allosteric inhibitor.

Compound **5**, covalently connected with active site cysteine of dimeric Mpro, was colored navy blue and red, and the purified structure of dimeric Mpro without small drugs was colored light blue and pink. In Figure 1b, the structure of dimeric Mpro is superimposed on each corresponding protomer part. The structure of the protein gradually expanded when bound to Compound **5** and Compound **2**. There was a distinct disorder that occurred in the extreme C-terminal region of Mpro linked to Compound **5**, suggesting conformational changes in several residues. As a direct comparison of the structural changes, the size of the motions induced by Compound **5** is larger than Compound **2**, which indicates that Compound **5** would disrupt the stability of Mpro conformation. Mpro with Compound **2** has a more stable configuration. The RMSF profiles further verified the results: the average value of RMSF for Mpro with Compound **5** is larger than that of Compound **2**, which are 0.176 nm and 0.162 nm (Figure S1a), respectively. Additionally, although Compound **5** and Compound **2** bound to dimeric Mpro all rendered the progressive increase of distance and angle between the C-terminal and N-terminal domains with simulation time, Compound **5** showed the most significant distance enhancements (Figure S2a,b). This correlated with the rotation angle and transient structure of monomeric Mpro discussed above. In essence, both catalytic and α-helical domains apparently shift from the active dimer Mpro to the protomer of dimeric Mpro, then to the compact monomer Mpro, and finally to the extended monomer Mpro. Hence, we speculated that Compound **5** bound to Mpro disrupted the loop-loop interactions between the C-terminal and N-terminal domains, leading to loss of contact with its own C-terminal domain. These observations indicate that Compound **5** can stabilize the monomeric extended conformation and prevent the formation of the compact monomer and active dimer Mpro, thereby serving as an allosteric enzyme inhibitor. In contrast, the transient conformation of dimeric Mpro bound to Compound **2** differs from bound to Compound **5**. The catalytic domain and helix domain surrounding Compound **2** exhibited slight expansion to accommodate the trifluoromethyl thiazole moiety. The amplitude of the upward movement of β-turn on inactive dimer Mpro connected with Compound **2** was smaller compared to that connected with Compound **5** (Figure 1c). Moreover, the distance and angle between the N-terminal and C-terminal domains remain relatively stable, which are lower than bound to Compound **5** (Figure S2a,b), suggesting that the dimerization of dimeric Mpro connected with Compound **2** was partially impaired.

### 3.2. Structural Stability of the Acrylamide Fragments Target SARS-CoV-2 Mpro

To assess the dynamic processes and binding stability of acrylamide fragments on SARS-CoV-2 Mpro, we utilized the root mean square deviation (RMSD) to track conformational stability during the 300 ns simulation. Typically, RMSD serves as a metric for dimerization stability, providing insights into the kinetics between biomolecules and nanodrugs [37]. We estimated the RMSD of the protein backbone of all these systems (Figure 2). The equilibrium period was 160–300 ns. The RMSD of dimeric Mpro with any Compound maintained a constant value (~0.25–0.37 nm) from 100 ns to the end of the MD simulation (Figure 2a). Obviously, the average RMSD values for Mpro with Compound **2** experienced a larger fluctuation compared to Mpro without any Compound, with a value of 0.26 ± 0.04 nm, indicating that Compound **2** caused comparative instability of the geometric structure of Mpro. In contrast, the average RMSD values of Mpro with Compound 5 was ~0.35 ± 0.05 nm, suggesting Compound **5** aggravates the instability of global conformation. Figure 2b shows the average RMSD values of monomeric Mpro maintained at ~0.35 ± 0.05 nm after 30 ns, and the average RMSD values for both Mpro with Compound 2 and Mpro with Compound **5** were 0.34 ± 0.05 nm and 0.37 ± 0.05 nm, respectively. This result induced by Compound **5** was consistent with dimeric Mpro and emphasized that Compound **5** would cause structure variations and transformations. Notably, the RMSD of active dimeric units showed less fluctuation compared to the Mpro monomer, indicating decreased stability of the monomeric Mpro structure relative to the dimeric one. This suggested Compound **5** could influence the stability of the target protein, especially for monomeric Mpro, thereby inducing significant conformational fluctuations and potentially disrupting the dimerization of active Mpro. Comparatively, the significant fluctuation of RMSD on monomeric Mpro compared to dimeric Mpro suggests that these fragments exhibited a stronger allosteric effect on monomeric Mpro than on dimeric Mpro.

**Figure 3:**
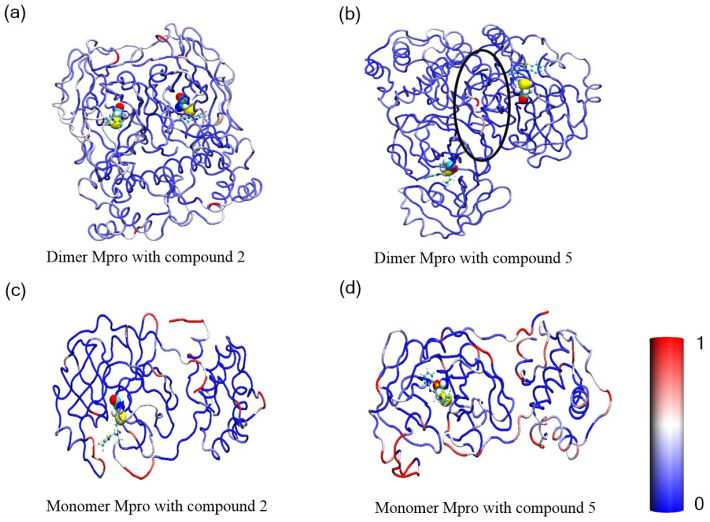
RMSD of each residue on dimeric Mpro covalently attached to (**a**) Compound **2** and (**b**) Compound **5**, and RMSD of each residue on monomeric Mpro covalently bound with (**c**) Compound **2** and (**d**) Compound **5**. The blue amino acid residues represent those with the smallest motion amplitude, while the red residues correspond to those with the largest motion amplitude.

To further evaluate both intrinsic and mutation-induced flexibility of active site residues, we also calculated the root mean square deviation (RMSD) for all amino acid residues within Mpro. The RMSD plots exhibited similar fluctuation patterns with varying magnitude across the simulated systems (see Figure 3). In this representation, red denotes highly active amino acid residues, blue indicates fewer active residues, and white falls between these two extremes. The redder the color, the more intense the displacement. On the active site cysteine, Mpro had much more active amide acid residues compared to Compound **2**, further confirming that Compound **5** exhibited a higher allosteric effect, thereby inducing structure dissociation (Figure 3a,b). Furthermore, the orientation of one monomer of dimer Mpro turned back, which aligned with the analysis of transient conformation for Compound **5** linked to monomeric Mpro (Figure 1a). Figure 3c,d illustrates the average RMSD per residue of monomeric Mpro covalently linked to Compound **2** and Compound **5**. As shown in Table 2, the RMSD results for both monomeric and dimeric forms of the proteins interacting with Compound **2** and Compound **5** show that the values are close when compared, indicating similar structural stability. However, by considering the standard error of the mean (SEM), which reflects the variability in RMSD across different residues, we can better understand the fluctuations. For Compound **2**, the RMSD of the monomer is 0.147 with an SEM of 0.030, while for the dimer, it is 0.139 with an SEM of 0.032. Similarly, for Compound **5**, the monomeric RMSD is 0.197 (SEM = 0.033), and the dimeric form has an RMSD of 0.184 (SEM = 0.016). These SEM values highlight the inherent fluctuations in RMSD across different residues, with the dimeric forms of both Compounds showing slightly lower variability in comparison to their monomeric counterparts. This suggests that while the overall structural deviations between monomeric and dimeric forms are comparable, the stability of individual residues varies more significantly in the monomeric forms. Particularly, the significant difference suggested that dimeric Mpro with Compound **5** experienced notably unstable fluctuation, further indicating that acrylamide 5 likely contributes to dimerization disruption.

### 3.3. Hydrogen Bond and Conformation Analysis for Acrylamide Fragments Covalently Connected with Active Site Cysteine of Mpro

**Figure 4:**
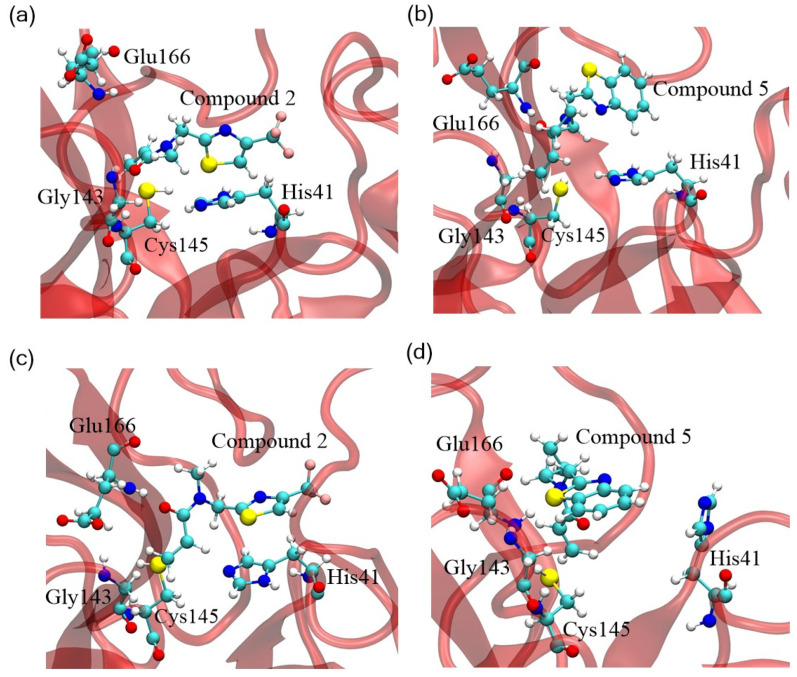
Amino acids within 0.50 nm of the active site cysteine Cys145 on dimeric Mpro with (**a**) Compound **2** and (**b**) Compound **5**, and on monomeric Mpro with (**c**) Compound **2** and (**d**) Compound **5**.

To explore the underlying mechanism of interaction between acrylamide fragments and SARS-CoV-2 Mpro, we predicted the binding configurations and relevant residue changes in both dimeric and monomeric Mpro. Further analysis of the affinity between biomolecules and nanomedicine was crucial in understanding their binding patterns and interaction details. Research has shown that the affinity of protein-drugs was driven by π-π stacks interaction, as well as weaker interactions such as hydrogen bonds and salt bridges, with a distance cutoff of 0.350 nm and an angle cutoff of 30° [38]. Hydrogen bonds play a significant role in stabilizing interactions within biological molecules [39]. Also, hydrogen bond occupancy was used to assess binding effects during molecular simulation [40]. For Compound **2**, a direct hydrogen bond was formed between oxygen 8 in the carbonyl group and the side chain nitrogen of Gly143. The hydrogen bond had a distance of 0.288 nm and an occupancy of 41.72% (Figure 4a), suggesting the potential electron transfer, which could influence the catalytic activity of acrylamide fragments. In contrast, Compound **5** exhibited an average hydrogen bond distance of 0.179 nm and an occupancy of 89.42%, indicating a stronger hydrogen bond formation with Glu166 (Figure 4b). Besides, the preference for π-π stacks and hydrophobic interactions via the aromatic ring (His41) and nonpolar side chains of the protein likely contributed to more stable binding interaction between Compound **5** and Mpro, undirectedly leading to the unfolding and dissociation of SARS-CoV-2 Mpro.

For acrylamide fragments covalently bonded to active site residue Cys145 of Mpro, we found Compound **5** anchored to the sulfur atoms of residue Cys145 and remained stable in the hydrophobic cavity within the catalytic domain (barrel fold). Especially, Compound **5** and Compound **2** exhibited similar binding patterns, enclosing themselves in a cavity formed by residues such as Cys145, Glu166, Gly143, and His41 (Figure 4a,b), whereby amino acids within 0.50 nm of the active site cysteine Cys145 were susceptible to Compounds **5** or **2**. These fragments interfered with the stability of Mpro by forming weak interaction and hydrophobic interaction with active site cysteine and surrounding amino acids, especially in the vicinity of the catalytic domain of Mpro. Most importantly, the catalytic dyad (comprising His41 and Cys145) within the barrel fold played a critical role. Residue Cys145, acting as a potent nucleophile, interacts with the amide backbone of the fragments, facilitating the formation of the oxyanion hole during the transition state, which is electrostatically stabilized [41]. The trifluoromethyl thiazole moiety of Compound **2** and the benzothiazole moiety of Compound **5** formed π-stacking interactions with the side chain of His41, further stabilizing these fragments (Figure 4a–d). With a distance of just 0.18 nm between the sulfur atom and the carbon atom in Cys145, the binding configuration 9 was likely influenced by the spatial positioning of the acrylamide fragments. Moreover, Compound **5** exhibited a unique inhibitory mechanism with its hydrophobic benzothiazole group and the exposed cyclopropane group. These features enlarged the substrate binding pockets and created space for the benzothiazole moiety, consequently affecting the surrounding residues. These results revealed that the structural stability of protein fragments was primarily driven by the strength of hydrogen bonds, followed by π-π stacks interactions, and then hydrophobic interactions between the fragments and Mpro. This evidence shows the destabilization of dimeric Mpro following covalent bonding with these two acrylamides, particularly Fragment 5.

In the case of Compound **2**-Mpro monomer complexes, the absence of hydrogen bonds between the fragments and Mpro monomers pointed to π-π stacks and hydrophobic forces as the primary stabilizing factors. These interactions inhibited the transition of the Mpro monomer into the protomer of the dimer (inactive dimer), as illustrated in Figure 4c, d. It was also noted that the stable binding of Compounds to dimeric Mpro led to a decrease in the total number of hydrogen bonds in Mpro homodimers (Figure S3a), as well as in monomeric Mpro (Figure S3b). Mpro connected with Compound **5** also had a significant decrease than when linked to Compound **2**. This suggested that the initial helical conformation of the protein was seriously disrupted when bound to Compound **5**. Furthermore, this evidence supported the above analysis that dimerization was partially impaired when bound to Compound **2**, which emphasized the crucial role of strong hydrogen bonds in small drugs-Mpro dimer complexes for maintaining active conformation.

### 3.4. The Interactions Between Mpro Protomers at Boundaries

**Figure 5:**
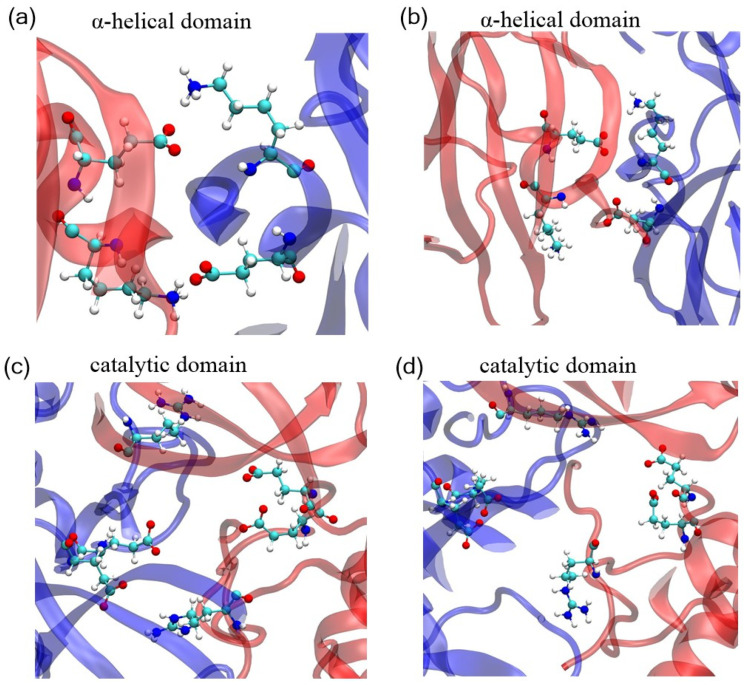
Interactions between chain A (red) and chain B (navy blue) of the helical domain connected with (**a**) Compound **2** and (**b**) Compound **5**. The interactions between chain A and chain B of the catalytic domain connected with (**c**) Compound **2** and (**d**) Compound **5**, respectively.

Ionic bonds, or salt bridges, are electrostatic interactions between negatively charged oxygen atoms of acidic residues and positively charged nitrogen atoms of basic residues when they are within 0.50 nm of each other [42]. Analyzing the statistical salt bridge interaction of inter-chain A-B at the boundary provides an effective strategy for exploring the conformational changes of Mpro [43]. For a detailed structural analysis, we divided dimeric Mpro into upper (α-helical domain) and lower regions (catalytic domain) connected with Compound **2** (Figure 5a,c) or Compound **5** (Figure 5b,d). Compared with the conformational interaction of α-helical domains of dimeric Mpro at boundaries, the protomers bound much more tightly when connected with Compound **2** than when linked to Compound **5** (Figure 5a,b). Besides, the binding distance among interaction amino acid pairs of Mpro connected with Compound **2** is lower than with Compound **5**, especially in the catalytic domain (Figure 5c,d). This suggests that Mpro with Compound **5** has a profound allosteric effect, whereby Compound **5** exhibited a higher allosteric target, causing a significant disruption of dimerization, thereby leading to better inhibitory enzyme activity. Moreover, the plots of global conformational interaction for interaction amino acid pairs were a supplement for catalytic domains and α-helical domains of dimeric Mpro at the boundaries (Figure S4a,b).

Furthermore, a salt bridge network was initially formed at the dimerization interface (Figure S5a,b). Further analysis of the binding distance of amino acid pairs confirmed that Compound **5** induced considerable dissociation of the catalytic or α-helical domain of Mpro. In Figure S5a,b, the α-helical domain of dimeric Mpro contains two weak hydrogen bonds and salt bridges between residue Glu14 and Lys12 of chain A-B bound to Compound **2**. In contrast, there were no weak interactions in the catalytic domain of Mpro linked to Compound **5**. The average distances between interaction amino acid pairs for Glu14-Lys12, Arg4-Glu288, and Arg4-Glu290 of chain A-B in Mpro with Compound **2** were 0.366 nm, 0.403 nm; 0.921 nm, 0.543 nm; and 0.743 nm, 0.317 nm, respectively (Figure S5a). However, in Mpro with Compound **5**, the corresponding average distances were 0.677 nm, 0.447 nm; 1.266 nm, 0.817 nm; and 1.065 nm, 0.807 nm (Figure S5b). The distance difference indicated a shift in inter-chains of Mpro, thereby leading to partial impairment of dimerization of Mpro with Compound **5**. Also, the catalytic dyad of Mpro, which was closer to the acrylamide fragments, is more easily disturbed in terms of spatial position. Notably, compared with Compound **2**, Compound **5** bound to dimeric Mpro resulted in a greater reduction in the number of salt bridges at boundaries (Figure S6a), and the contact distance among key residues of chain A-B increased more significantly (Figure S6b). This suggests that there is a greater displacement movement between dimeric units. These observations suggest that acrylamide fragments can disrupt the dimerization of Mpro, causing substantial distortion in the orientation and distance between the N-terminal and C-terminal domains, especially protein linked to Compound **5**. Therefore, Compound **5** exhibited a better binding affinity with SARS-CoV-2 Mpro compared to Compound **2**. Therefore, Compound **5** had a stronger inhibitory effect against Mpro and can be considered an allosteric inhibitor.

### 3.5. Comparison of Inhibitory Effect of Acrylamide Fragments on Mpro

**Figure 6:**
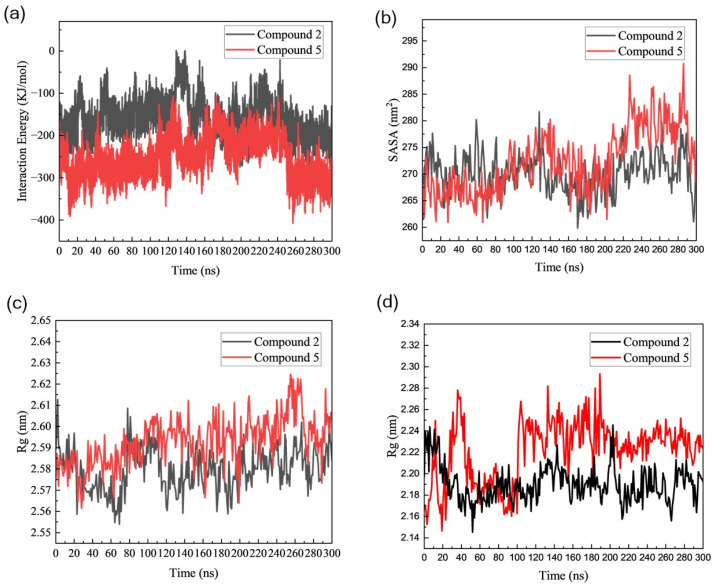
Time-series analysis of allosteric effect for SARS-CoV-2 dimeric or monomeric Mpro. (**a**) The interaction energy of chain A-B in Mpro dimers after bound to Compound **2** and Compound **5**. (**b**) Solvent accessible surface area (SASA) values of Mpro dimers. (**c**) Radius of gyration (Rg) analysis of Mpro dimers. (**d**) Rg values of Mpro monomers.

To evaluate the inhibitory efficacy of acrylamide fragments on Mpro, we conducted a comprehensive analysis of various structural properties, including radius of gyration (Rg), solvent-accessible surface area (SASA), and interaction energy, for both Mpro dimers and monomers during 300 ns simulation. Non-bonded interactions, specifically van der Waals interaction and electrostatic energy measured in terms of Coulomb and Lennard-Jones (LJ) potential functions, play a pivotal role in interaction energy [43]. We observed that the interaction energy between Mpro dimers and Compound **2** decreased to an average value of −160.62 ± 43.92 KJ/mol. Conversely, Mpro linked with Compound **5** exhibited lower binding free energy, with an average value of −252.74 ± 46.56 KJ/mol. Besides, this interaction energy decreased even more dramatically, reaching a value of −407.65 KJ/mol at 260 ns (Figure 6a). To further quantify the binding affinities of the fragments on Mpro, the calculations of binding free energies were performed using the last 10 ns of MD trajectories. The binding free energy values of Mpro-Compound **2** were found to be −74.74 KJ/mol, whereas, for Mpro-Compound **5** complex, it was −182.22 KJ/mol. Further MM-PBSA analysis indicated that Compound **5** binding reduced the interaction energy between Mpro monomers to 405.04 kJ/mol, compared to 445.46 kJ/mol for Compound **2**. In contrast, the Mpro dimer without any Compound binding had an interaction energy of −487.64 kJ/mol. These results suggest that covalent bonding with either Compound **2** or Compound **5** weakens the affinity between Mpro monomers, with Compound **5** having a particularly destabilizing effect on the dimeric complex. Throughout the simulation, Compound **5** induces progressive destabilization at the dimer interface, leading to partial dissociation of the Mpro dimer. This structural disruption may contribute to a reduction in the catalytic activity of Mpro (Table 3).

Additionally, we calculated the solvent-accessible surface area (SASA) to assess the extent of expansion of the Mpro dimer volume. The average SASA values for Mpro bound to Compound **2** and Compound **5** were 270.11 ± 3.15 nm^2^ and 272.24 ± 5.95 nm^2^, respectively (Figure 6b). This suggests that Compound **2** caused a slighter expansion in the Mpro dimer. Moreover, we used the radius of gyration (Rg) to evaluate the compactness of Mpro after acrylamide fragments covalently bound to the active site residue Cys145 in both Mpro dimers and monomers. The average Rg value for dimeric Mpro with Compound **5** was 2.60 ± 0.01 nm, while it was slightly lower for dimeric Mpro with Compound **2**, at 2.57 ± 0.01 nm, indicating that Mpro dimer with Compound **5** is less compact compared to Mpro with Compound **2** (Figure 6c). Similarly, we analyzed the Rg values of Mpro monomers, where the average Rg value for monomeric Mpro bound to Compound **2** was 2.19 ± 0.02 nm, while there was a slight increase for monomeric Mpro bound to Compound **5**, with an average Rg value of 2.23 ± 0.03 nm (Figure 6d). These results confirmed Mpro connected with Compound **5** had a looser conformation compared to that connected with Compound **2**. Particularly, Mpro dimers are less compact than Mpro monomers. This indicated that the initial protein helical conformation is disrupted by the binding of acrylamide fragments. Therefore, Compound **5** specifically targeted the dimeric Mpro, locking it in its monomeric form. These findings further indicate that Compound **5** is a potential allosteric inhibitor for the enzyme and could be a promising drug candidate against COVID-19 or viruses resembling coronavirus.

**Figure 7:**
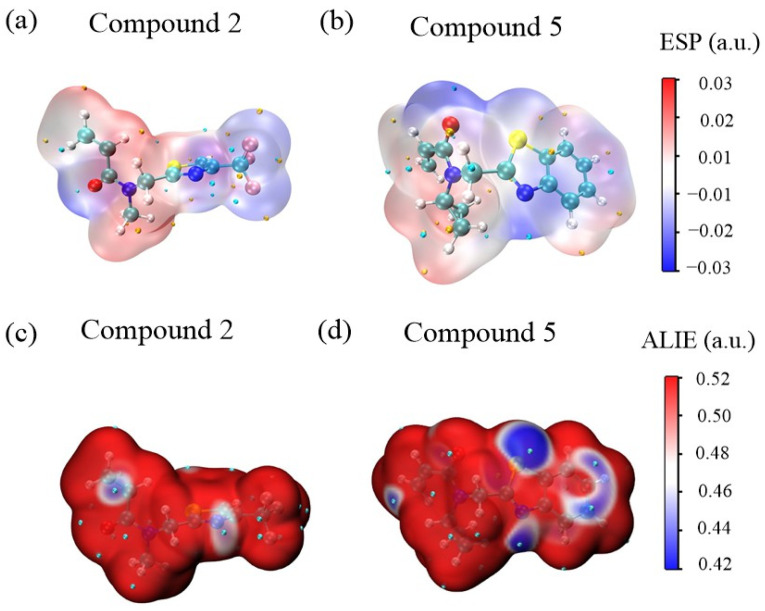
(**a**,**b**) Electrostatic surface potential (ESP) analysis for different Compounds. (**c**,**d**) Average local ionization energy (ALIE) mapped onto the van der Waals surface of the Compounds. The blue regions, indicating weaker electron density, suggest higher reactivity of the electrons in the acrylamide fragments, making these areas more susceptible to electrophilic reactions.

The molecular electrostatic potential (ESP) [44] and average local ionization energy (ALIE) maps for Compound **2** and Compound **5** provide insight into their potential interactions with SARS-CoV-2 Mpro. In the ESP maps (Figure 7a,b), blue regions indicate areas of negative electrostatic potential, where the electron density is high, while red regions represent positive potential, indicating electron-deficient areas. For Compound **5**, negative charge density is concentrated around oxygen, nitrogen, and sulfur atoms, while positive charge density is mainly located near the cyclopropane and adjacent portions, with the aromatic ring appearing neutral. This distribution suggests stronger nucleophilic potential in Compound **5**, enhancing its affinity for electrophilic interactions with Mpro. For Compound **2**, negative charge density is concentrated around the oxygen and trifluoromethyl groups, with the rest of the molecule showing positive charge density. The introduction of the thiazole ring in Compound **5** increases the electrophilicity of the molecule, which may further contribute to its enhanced binding affinity. In the ALIE maps (Figure 7c,d), blue areas indicate regions where electrons are weakly bound and more likely to participate in reactions. Compound **5** displays larger blue regions and more cyan spheres (indicating ALIE minima), suggesting that it has more reactive sites and a greater likelihood of undergoing electrophilic attacks. In contrast, Compound **2** shows fewer reactive areas. These differences indicate that Compound **5** has a stronger binding potential and reactivity compared to Compound **2**, supporting its superior inhibitory ability against Mpro.

### 3.6. Determination of Structure Variation of SARS-CoV-2 Mpro upon PCA-Based 2D Free-Energy Surfaces

**Figure 8:**
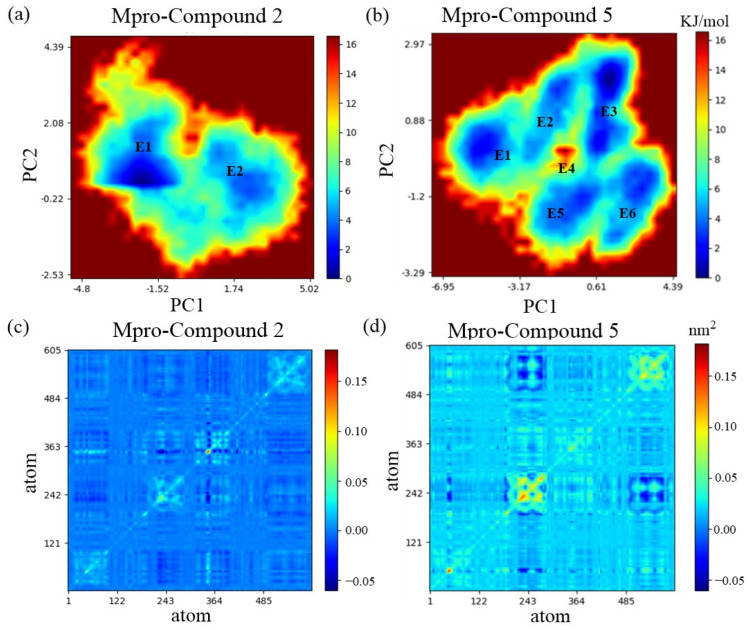
Comparative analysis of Mpro from PCA and covariance matrix. The conformational space sampled of MD trajectories projected onto subspaces spanned by PC1-2 of the Mpro complex with (**a**) Compound **2** and (**b**) Compound **5**, respectively. Covariance matrix calculations in atomic coordinates corresponding to Mpro complexed with (**c**) Compound **2** and (**d**) Compound **5**, respectively.

A Gibbs free energy landscape (FEL) [45,46] was used to explore the global structure dynamics changes of Mpro bound to Compound **2** and Compound **5** on the conformational space. Two principal components analysis (PCA) referencing PC1 and PC2 reaction coordinates reflect the principal modes of structural variation, which were obtained from C atomic fluctuation. It is observed that the collective motion of Mpro connected with Compound **2** is located on two main energy basins, E1 and E2, representing different conformational stability of Mpro Compounds (Figure 8a). They signify that the structure of Mpro is clustered into two clusters (Figure S7a). The color of E1 is dark blue than the color of E2, which suggests steady conformation. The twisting motion of the structure of Mpro with Compound **2** occurs in the E2 subspace. Also, the magnitude of motion along PC1 is comparable to the size of PC2 at 1 5 KJ/mol. In comparison to Compound **2**, the structure fluctuations of Mpro with Compound 5 navigate a broader conformation space, with the configurations primarily distributed in six energy basins, from E1 to E6 (Figure 8b). Notably, Mpro with Compound **5** caused a clear separation of the structures into six clusters (Figure S7b), demonstrating global conformations of Mpro that generated evident deviation from each other. Hence, the fewer the clusters, the less flexible the protein, and the structure distribution and flexibility of Mpro with Compound **2** is confined to a small conformational space. Furthermore, E4 has the highest energy values of 12 15.6 KJ/mol compared to the other five energy wells, suggesting significant torsion and displacement, thereby possibly disrupting the secondary structures of Mpro and influencing the enzyme activity. Notably, PC1 shows larger movement of the thumb on structure variation.

Furthermore, the corresponding covariance matrices (also called dynamics cross-correlation maps—DCCM) were built to describe the mean-square deviations in atomic coordinates from their mean position [47] or the coupled linear motion correlations between their pairwise fluctuations [48]. The native values represent relative motions, and the positive values represent synchronous movements. The larger the positive value, the more unstable the conformation. In cross-correlation maps (CCij), Mpro bound to Compound **5** generates more coupled motions between residues (Figure 8c) compared to that bound to Compound **2** (Figure 7d), which indicates obvious instability of the Mpro structure. Particularly, residues 201 through 213 of the helix of Mpro with Compound **5** show the most coupled motions (Figure 8d), and some residues in the domain play a pivotal role in maintaining the activity of the main protease, demonstrating the instability of the helix domain. The secondary structure of Mpro with Compound **5** has transformed from a helix into a coil, which indicates a non-secondary structure area (Figure S7d). By contrast, the secondary structure of Mpro with Compound **2** has changed from helix to turn partly (Figure S7c). These results were consistent with the 2D free energy surface and the interaction energy analysis (Figure 6a). Therefore, the binding of Compound **5** exhibited greater inhibitory function on SARS-CoV-2 Mpro.

Overall, the MD simulation results are consistent with the experimental findings, further confirming that Compound **5** causes the main protease to elute as a monomer, while Compound **2** causes the main protease to elute as a dimer. Compared with traditional active-site inhibitors, the mechanism in our study involves acrylamide fragments targeting the active-site cysteine Cys145 and occupying the substrate-binding pocket, leading to conformational distortions. These distortions affect the active site and surrounding regions, inducing transitions between different conformational states (active dimer, inactive compact dimer, and extended monomer), ultimately disrupting dimerization by releasing the constraints between the C-terminal and N-terminal regions of Mpro. However, C-terminal and N-terminal regions play a pivotal role in the dimerization and enzyme activity of SARS-CoV-2 Mpro. Therefore, allosteric inhibitors such as Compound **5** may offer certain advantages; they can induce conformational changes at sites distant from the active site, potentially reducing the emergence of drug resistance, as these sites are less prone to mutations under selective pressure. Additionally, by targeting alternative regions, allosteric inhibitors could complement the effects of active-site inhibitors, possibly enhancing therapeutic efficacy [49,50,51]. Additionally, the molecular-level mechanism of the antiviral effect of the acrylamide fragment is revealed, showing that Compound **5** disrupts the secondary structure of the protease, significantly affecting its dimerization and impairing viral activity, implying substantial opportunities for the development of benzothiazole core Compounds.

## 4. Conclusions

In this study, we identify a set of novel nanodrugs against SARS-CoV-2 Mpro, then construct dimeric and monomeric Mpro structures with two different Compounds to explore their underlying inhibitory mechanism of interaction from the molecular level. The two nanodrugs trapped distinct binding conformation of Mpro from an active dimeric to an inactive monomeric state. Complemented with previously reported experiments [20], these results confirmed that acrylamide fragments destabilized the dimerization of Mpro. Particularly, we determined the therapeutics with the covalent mechanism of acrylamide fragments from the molecular level. Besides, compared with Compound **2**, Compound **5** exhibits a superior inhibitory efficacy when targeting Mpro, whether with monomers or dimers. The substitution of trifluoromethyl and methane with aromatic benzene and cyclohexane moieties, respectively, is suggested to be responsible for the enhanced binding potential of Compound **5**. The binding ability is driven by a larger number of hydrogen bonds and salt bridges between Compound **5** and key residues around the catalytic cysteine (Cys145), such as Glu166 and Gly143. Especially, we observed that acrylamide fragments covalently bound to catalytic cysteine Cys145 would disrupt the interaction between the catalytic domain and the helix domain, leading to gradual dissociation of protein structure, thereby inhibiting the autocleavage of Mpro and suppressing enzyme activity. The continuous dissociation of N- and C-terminals on monomeric Mpro interfered with the enzyme maturation from the early stage. Therefore, the acrylamide fragment is specific to the monomeric Mpro, inhibiting the transformation of monomeric to dimeric Mpro.

The current work may shed light on the substantial potential for the development of small molecular drugs with acrylamide warheads, especially Compound **5**. Most importantly, our study reveals an allosteric target by elucidating binding-induced structural conformational changes, and the theoretical complement for experiments provided more precise predictions for acrylamide fragments inhibiting Mpro. Our data offers detailed insights into the molecular inhibition mechanism of acrylamide fragments, complementing the available literature on fragment-based drug design targeting Mpro. Recently, various structural modifications to acrylamide warheads resulted in fluorinated acrylamide and cyano-acrylamide warheads, among others [52]. These small molecules have been widely applied in kinase inhibitors and antiviral drugs. Their therapeutic effects and targeting mechanisms demonstrate the positive impact of acrylamide warheads on drug development. Overall, our findings contribute to this growing body of knowledge by providing a molecular-level understanding of acrylamide’s role in inhibiting viral proteases, which could guide and accelerate the rational design of more effective Mpro inhibitors.

## Figures and Tables

**Table 1:** Molecular structure of Compound **2** and Compound **5** [20].

System	Compound 2	Compound 5
Molecular structure	** 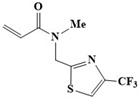 **	** 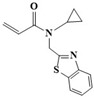 **

**Table 2:** The average RMSD and SEM of amino acid residue of protein in both dimeric and monomeric Mpro.

RMSD/nm	Dimer	Monomer	SEM (Dimer)	SEM (Monomer)
Compound **2**	0.139	0.147	0.032	0.030
Compound **5**	0.184	0.197	0.016	0.033

**Table 3:** MM-PBSA values of different Mpro systems.

System	Binding Free Energy (KJ/mol)
Mpro-Compound **2**	−74.74
Mpro-Compound **5**	−182.22
Monomers chainA-chainB under Compound **5**	−405.46
Monomers chainA-chainB under Compound **2**	−445.04
Monomers chainA-chainB without ligand	−487.64

## Data Availability

Data is contained within the article and Supplementary Materials.

## Supplementary Materials

The following supporting information can be downloaded at: https://www.mdpi.com/article/10.3390/cimb46110765/s1. Figure S1: RMSF profiles of Mpro bound to Compound 2 and Compound 5. The RMSF value of dimeric Mpro(a) and monomeric Mpro(b); Figure S2: (a, b) The distances and angles between the C-terminal and N-terminal regions of dimeric Mpro connected with Compound 2 and Compound 5; Figure S3: (a) Hydrogen bonds between chain A and B of dimeric Mpro bound to Compound 2 and Compound 5. (b) Hydrogen bonds between monomeric Mpro and Compound 2 or Compound 5; Figure S4: Global interaction patterns of amino acid pairs of dimeric Mpro connected with (a) Compound 2 and (b) Compound 5; Figure S5: The average interaction distances of dimeric Mpro at the dimer boundaries (Å), covalently attached to (a) Compound 2 and (b) Compound 5; Figure S6: (a)The salt-bridges of dimeric Mpro connected with Compound 2 and Compound 5 at boundary. (b)The contact distances (nm) among interacting amino acid pairs of dimeric Mpro linked to Compound 2 and Compound 5 at the boundaries; Figure S7: The sites and amounts of Mpro structure clusters induced by (a) Compound 2 and (b) Compound 5. The transient conformation of SARS-CoV-2 Mpro connected with(c) Compound 2 and (d) Compound 5; Figure S8: RMSD analysis of the Mpro-Compound complexes. A smaller RMSD vale indicates a more stable binding of Mpro-Compound complexes. The equilibrium state is achieved by 100 ns with conformational fluctuations remaining below 0.5 Å. Notably Compound 5 exhibits greater binding stability with Mpro compared to Compound 2; Figure S9: Analyzing of ESP and ALIE of Compound 5-Cys145 or Compound 2-Cys145; Table S1: List of structure properties for dimeric and monomeric Mpro without Compounds; Table S2: List of structure properties for monomeric Mpro with Compound 2 and Compound 5; Table S3: List of structure properties for dimeric Mpro with Compound 2 and Compound 5; Table S4: List of atomic charge, bonding, angles, and dihedrals for Compound-Cys145.
