# Supplementary material for: Computational Insights into Acrylamide Fragment Inhibition of SARS-CoV-2 Main Protease
Source: Curr Issues Mol Biol. 2024 Nov 12;46(11):12847–65. doi: 10.3390/cimb46110765 (PMC11592536; doi:10.3390/cimb46110765)
Supplement: Supplementary file 1 [file cimb-46-00765-s001.zip › cimb-3278039-supplementary.pdf]

Supporting Information for

Computational Insights into Acrylamide Fragment Inhibition of SARS-CoV-2 Main Protease

Ping Chen[†1 2] Liyuan Wu[†1] Bo Qin[3 4] Haodong Yao[1] Deting Xu[1] Sheng Cui[3 4] and Lina Zhao [1 2*]

[1] CAS Key Laboratory for Biomedical Effects of Nanomaterials and Nanosafety, Institute of High Energy Physics, Chinese Academy of Sciences, Beijing 100049, China; chenping@ihep.ac.cn (P.C.); wuly@ihep.ac.cn (L.W.); yaohd@ihep.ac.cn (H.Y.); xudt@ihep.ac.cn (D.X.)

[2] University of Chinese Academy of Sciences, Beijing 100049, China

[3] NHC Key Laboratory of Systems Biology of Pathogens, Institute of Pathogen Biology, Chinese Academy of Medical Sciences & Peking Union Medical College, Beijing 100730, China; qbone@163.com (B.Q.); cui.sheng@ipbcams.ac.cn (S.C.)

[4] Key Laboratory of Pathogen Infection Prevention and Control (Peking Union Medical College), Ministry of Education, Beijing 100730, China

* Correspondence: linazhao@ihep.ac.cn; Tel.: +86-010-88238542

† These authors contributed equally to this work.

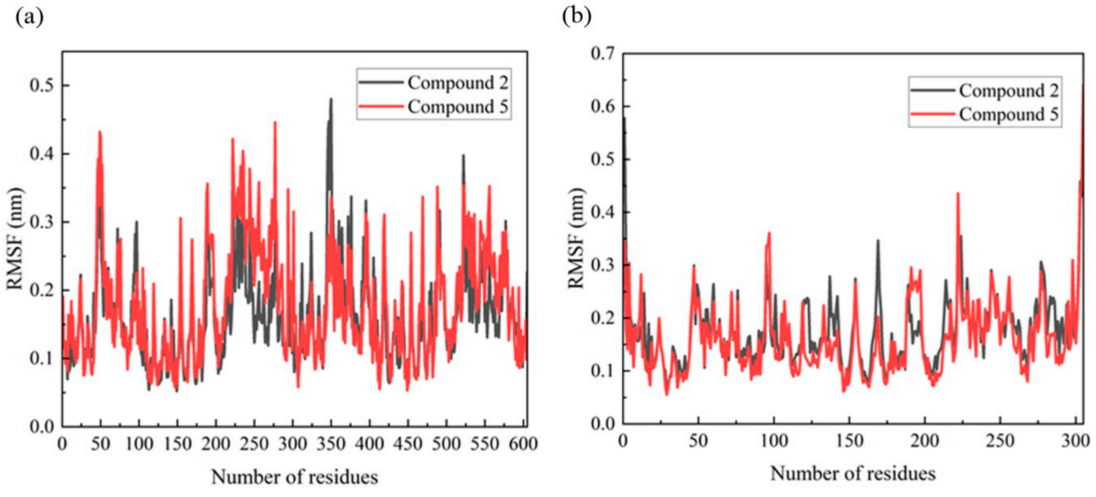

Figure S1. RMSF profiles of Mpro bound to Compound 2 and Compound 5. The RMSF value of dimeric Mpro(a) and monomeric Mpro(b).

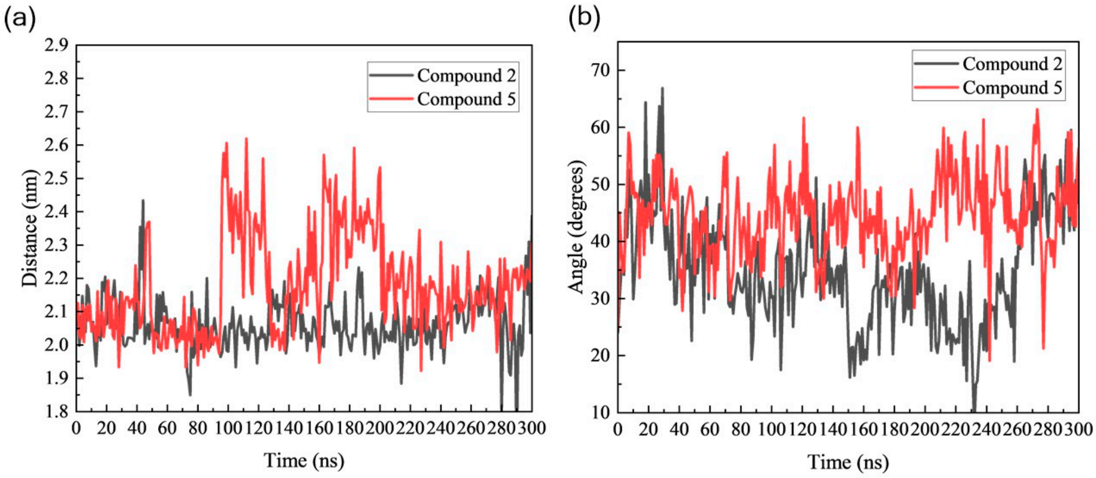

Figure S2. (a b) The distances and angles between the C-terminal and N-terminal of dimeric Mpro connected with Compound 2 and Compound 5.

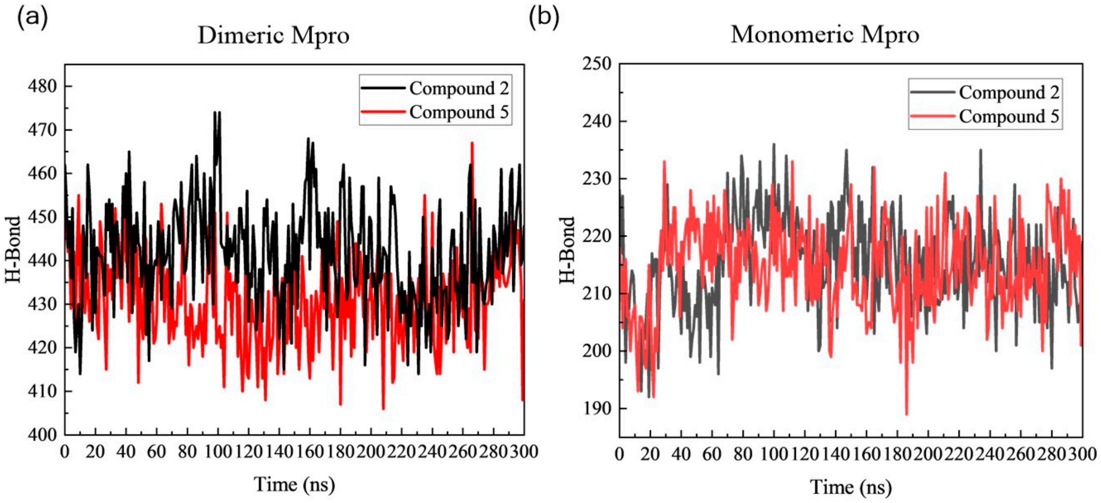

Figure S3. (a) Hydrogen bonds between chain A and B of dimeric Mpro bound to Compound 2 and Compound 5. (b) Hydrogen bonds between monomeric Mpro and Compound 2 or Compound 5.

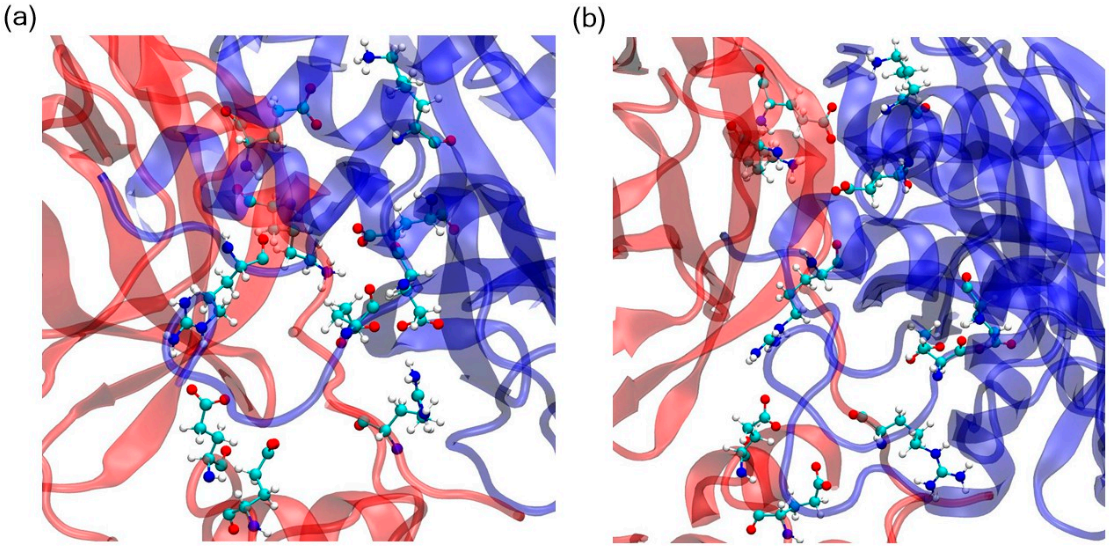

Figure S4. Global interaction patterns of amino acid pairs of dimeric Mpro connected with (a) Compound 2 and (b) Compound 5.

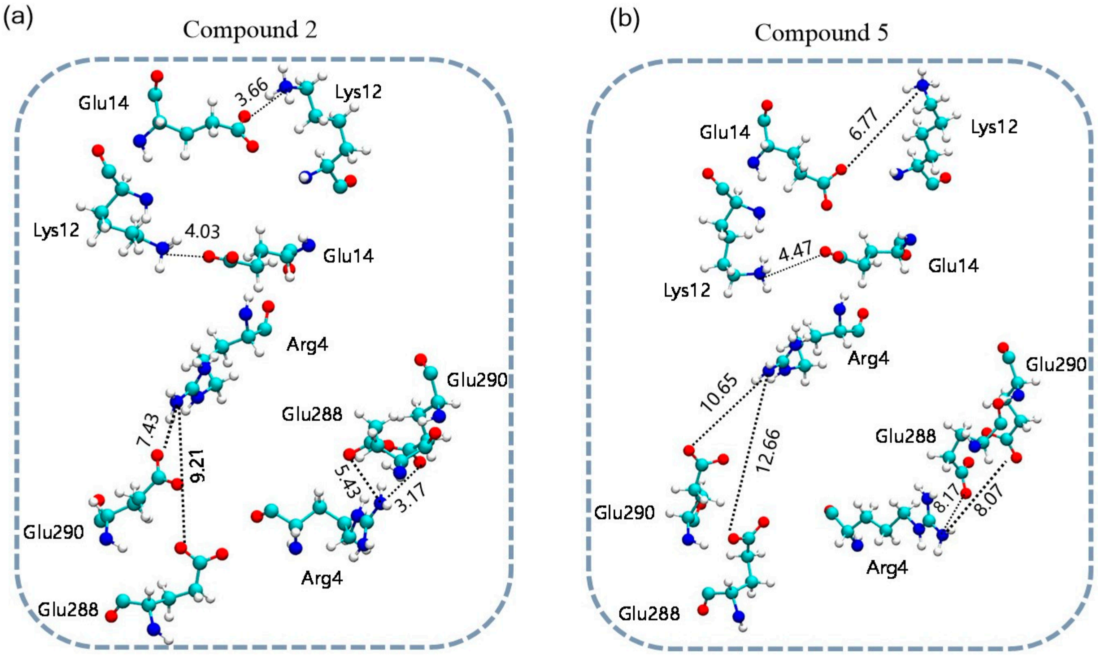

Figure S5. The average interaction distances of dimeric Mpro at the dimer boundaries (Å), covalently attached to (a)Compound 2 and (b) Compound 5.

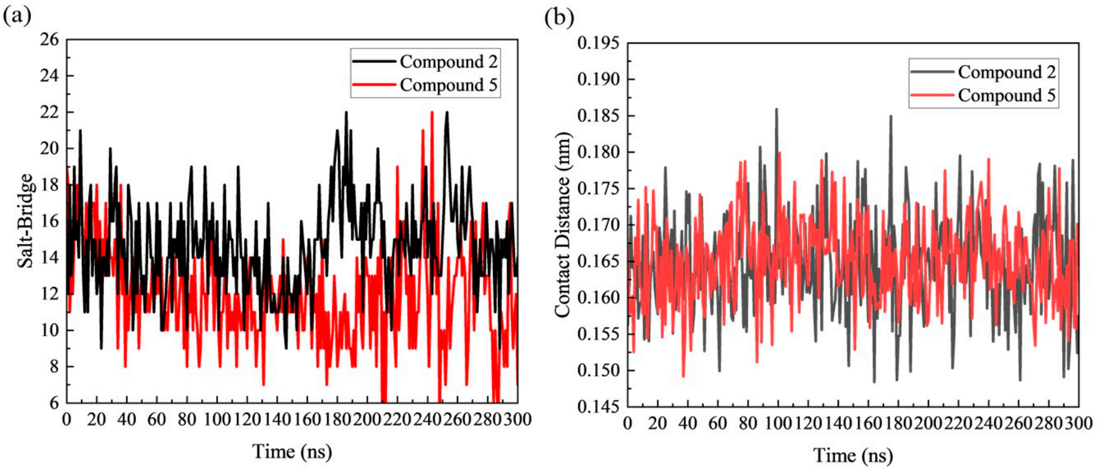

Figure S6. (a)The salt-bridges of dimeric Mpro connected with Compound 2 and Compound 5 at boundary. (b)The contact distances (nm) among interacting amino acid pairs of dimeric Mpro linked to Compound 2 and Compound 5 at the boundaries.

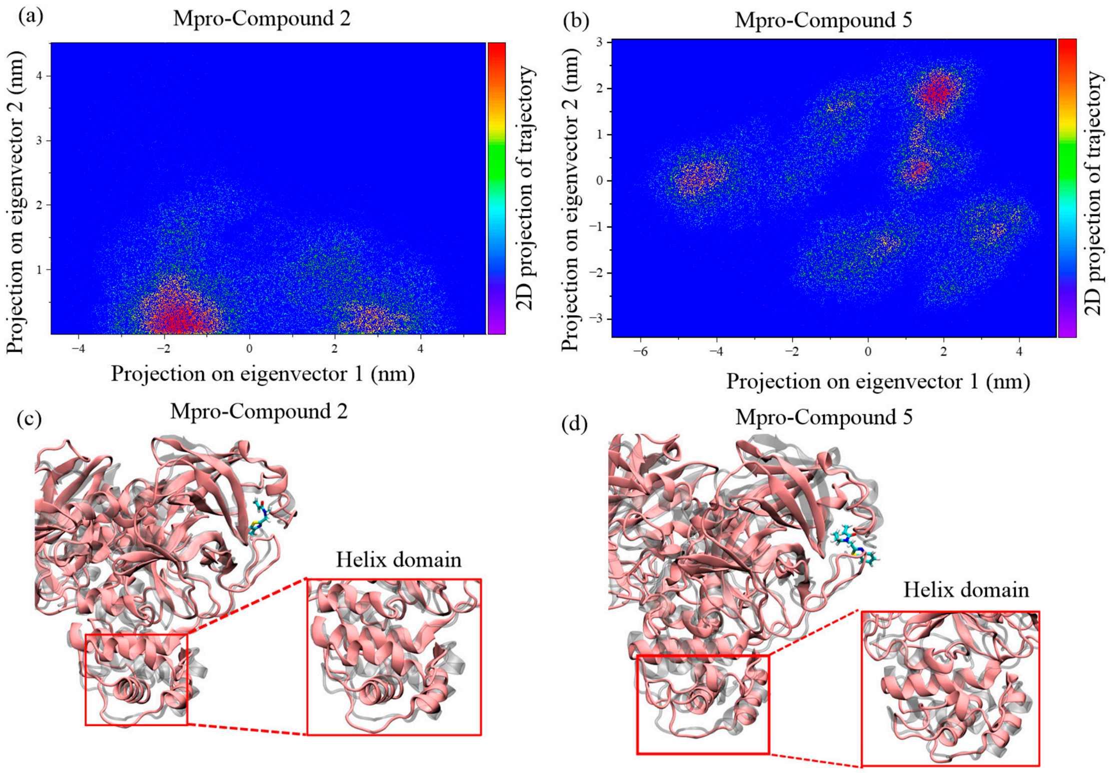

Figure S7. The sites and amounts of Mpro structure clusters induced by (a) Compound 2 and (b) Compound 5. The transient conformation of SARS-CoV-2 Mpro connected with(c) Compound 2 and (d) Compound 5.

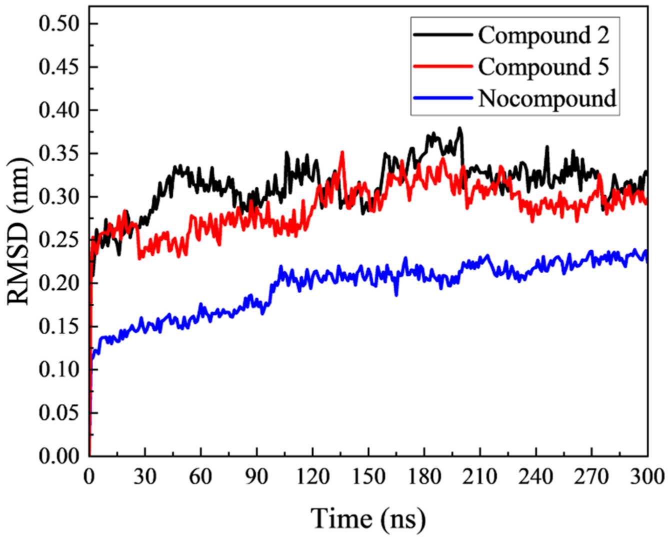

Figure S8. RMSD analysis of the Mpro-Compound complexes. A smaller RMSD vale indicates a more stable binding of Mpro-Compound complexes. The equilibrium state is achieved by 100 ns with conformational fluctuations remaining below 0.5 Å. Notably Compound 5 exhibits greater binding stability with Mpro compared to Compound 2.

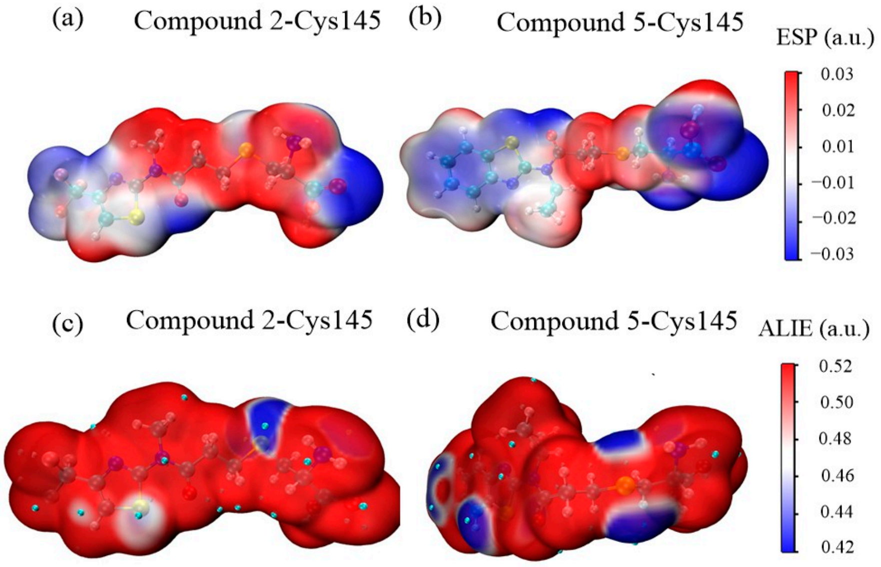

Figure S9. Analyzing of ESP and ALIE of Compound 5-Cys145 or Compound 2-Cys145.

Supplementary Table S1. List of structure properties for dimeric and monomeric Mpro without Compounds.

System	Molecular Dynamics	RMSD (nm)	Rg (nm)	SASA (nm^2)	RMSF (nm)
Dimer	MD_1	0.23	2.56	260.00	0.17
	MD_2	0.15	2.57	265.00	0.11
	MD_3	0.28	2.56	263.00	0.13
	Average	0.22	2.56	262.67	0.14
Monomer	MD_1	0.13	2.20	147.00	0.13
	MD_2	0.19	2.21	147.00	0.18
	MD_3	0.15	2.21	150.00	0.15
	Average	0.16	2.21	148.00	0.15

Supplementary Table S2. List of structure properties for monomeric Mpro with Compound 2 and Compound 5.

Compound	No.	RMSD (nm)	Rg (nm)	SASA (nm2)	RMSF (nm)	H-Bond	Interaction Energy (KJ/mol)
2	MD1	0.34	2.19	150.00	0.16	1	-99.08
	MD2	0.32	2.21	152.00	0.18	0	-47.91
	MD3	0.38	2.17	151.00	0.19	1	-47.91
	Average	0.35	2.19	151.00	0.18	0.7	-64.97
5	MD1	0.37	2.23	153.00	0.18	1	-111.74
	MD2	0.38	2.25	155.00	0.20	1	-102.51
	MD3	0.39	2.22	154.00	0.22	1	-88.11
	Average	0.38	2.23	154.00	0.20	1	-100.79

Supplementary Table S3. List of structure properties for dimeric Mpro with Compound 2 and Compound 5.

Compound	No.	RMSD (nm)	Rg (nm)	SASA (nm2)	RMSF (nm)	H-Bond	Interaction Energy (KJ/mol)
2	MD1	0.27	2.57	270.11	0.14	1	-160.62
	MD2	0.24	2.59	267.40	0.11	1	-174.31
	MD3	0.25	2.56	264.40	0.12	1	-218.21
	Average	0.25	2.57	267.30	0.12	1	-184.38
5	MD1	0.35	2.59	272.24	0.15	1	-252.74
	MD2	0.27	2.60	271.70	0.13	1	-232.75
	MD3	0.26	2.58	269.50	0.13	1	-247.64
	Average	0.29	2.59	271.15	0.14	1	-244.38

Supplementary Table S4. List of atomic charge, bonding, angles, and dihedrals for Compound-Cys145.

Compound 5-Cys145								Compound 2-Cys145							
atom	charge	bond	r0 (nm)	angles	a0 (Deg.)	dihedrals	d0 (Deg.)	atom	charge	bond	r0 (nm)	angles	a0 (Deg.)	dihedrals	d0 (Deg.)
C1	0.387612	C1-O2	0.12183	O2-C1-N3	123.05	C1-N3-C4-C5	0	C1	0.34829574	C1-O2	0.12183	O2-C1-N3	123.05	C1-N3-C4-N7	180
O2	-0.40169	C1-N3	0.13789	O2-C1-C41	123.2	C1-N3-C4-C6	0	O2	-0.4701133	C1-N3	0.13789	O2-C1-C34	123.2	C1-N3-C4-S8	180
N3	-0.12082	C1-C41	0.15241	N3-C1-C41	115.18	C1-N3-C4-H7	0	N3	-0.187228	C1-C34	0.15241	N3-C1-C34	115.18	C1-N3-C13-H14	0
C4	-0.14115	N3-C4	0.1434	C1-N3-C4	122.15	C1-N3-C12-N23	180	C4	0.3469303	N3-C4	0.13807	C1-N3-C4	123.27	C1-N3-C13-H15	0
C5	-0.26944	N3-C12	0.13807	C1-N3-C12	123.27	C1-N3-C12-S24	180	C5	0.14945319	N3-C13	0.14619	C1-N3-C13	120.69	C1-N3-C13-H16	0
C6	-0.23582	C4-C5	0.1508	C4-N3-C12	120.69	C1-C41-C38-S25	0	C6	-0.3294197	C4-N7	0.13694	C4-N3-C13	120.85	C1-C34-C31-S18	0
H7	0.175542	C4-C6	0.1508	N3-C4-C5	119.02	C1-C41-C38-H39	0	N7	-0.3902081	C4-S8	0.17562	N3-C4-N7	126.23	C1-C34-C31-H32	0
H8	0.136818	C4-H7	0.1089	N3-C4-C6	119.02	C1-C41-C38-H40	0	S8	0.07922576	C5-C6	0.14278	N3-C4-S8	122.88	C1-C34-C31-H33	0
H9	0.136818	C5-C6	0.1508	N3-C4-H7	114.26	O2-C1-N3-C4	180	C9	0.38715644	C5-N7	0.13694	N7-C4-S8	122.64	O2-C1-N3-C4	180
H10	0.12434	C5-H8	0.1087	C5-C4-C6	60	O2-C1-N3-C12	180	F10	-0.1422883	C5-C9	0.15015	C6-C5-N7	121.98	O2-C1-N3-C13	180

H11	0.12434	C5-H9	0.1087	C5-C4-H7	118.69	O2-C1-C41-C38	180	F11	-0.1575489	C6-S8	0.17562	C6-C5-C9	115.97	O2-C1-C34-C31	180
C12	0.305322	C6-H10	0.1087	C6-C4-H7	118.69	O2-C1-C41-H42	0	F12	-0.1391271	C6-H17	0.10817	N7-C5-C9	120.95	O2-C1-C34-H35	0
C13	0.185278	C6-H11	0.1087	C4-C5-C6	60	O2-C1-C41-H42	0	C13	-0.1051605	C9-F10	0.13497	C5-C6-S8	120.21	O2-C1-C34-H35	0
C14	0.038326	C12-N23	0.13694	C4-C5-H8	117.69	O2-C1-C41-H42	180	H14	0.07904696	C9-F11	0.13497	C5-C6-H17	127.96	O2-C1-C34-H35	180
C15	-0.1991	C12-S24	0.17562	C4-C5-H9	117.69	O2-C1-C41-H43	0	H15	0.07904696	C9-F12	0.13497	S8-C6-H17	119.97	O2-C1-C34-H36	0
C16	-0.1967	C13-C14	0.13984	C6-C5-H8	117.69	O2-C1-C41-H43	0	H16	0.07904696	C13-H14	0.10969	C4-N7-C5	103.76	O2-C1-C34-H36	0
C17	-0.08775	C13-C15	0.13984	C6-C5-H9	117.69	O2-C1-C41-H43	180	H17	0.22887944	C13-H15	0.10969	C4-S8-C6	90.24	O2-C1-C34-H36	180
H18	0.131028	C13-N23	0.13517	H8-C5-H9	114.43	N3-C1-C41-C38	0	S18	-0.2143998	C13-H16	0.10969	C5-C9-F10	111.31	N3-C1-C34-C31	0
C19	-0.15738	C14-C16	0.13984	C4-C6-C5	60	N3-C1-C41-C38	0	C19	-0.0509991	S18-C19	0.18392	C5-C9-F11	111.31	N3-C1-C34-C31	0
H20	0.178986	C14-S24	0.17806	C4-C6-H10	117.69	N3-C1-C41-H42	180	C20	0.24004179	S18-C31	0.18392	C5-C9-F12	111.31	N3-C1-C34-H35	180
H21	0.116225	C15-C17	0.13984	C4-C6-H11	117.69	N3-C1-C41-H43	180	H21	0.08892251	C19-C20	0.15375	F10-C9-F11	107.36	N3-C1-C34-H36	180
H22	0.124341	C15-H18	0.1086	C5-C6-H10	117.69	N3-C4-C5-C6	0	H22	0.08892251	C19-H21	0.10969	F10-C9-F12	107.36	N3-C4-N7-C5	180
N23	-0.3166	C16-C19	0.13984	C5-C6-H11	117.69	N3-C4-C5-H8	0	H23	-0.0152059	C19-H22	0.10969	F11-C9-F12	107.36	N3-C4-S8-C6	0

S24	-0.08112	C16-H20	0.1086	H10-C6-H11	114.43	N3-C4-C5-H9	0	N24	-0.7987527	C20-H23	0.10969	N3-C13-H14	108.88	C4-N3-C1-C34	180
S25	-0.21505	C17-C19	0.13984	N3-C12-N23	126.23	N3-C4-C6-C5	0	C25	0.59023006	C20-N24	0.14647	N3-C13-H15	108.88	C4-N3-C13-H14	0
C26	-0.28759	C17-H21	0.1086	N3-C12-S24	122.88	N3-C4-C6-H10	0	H26	0.32937629	C20-C25	0.15241	N3-C13-H16	108.88	C4-N3-C13-H15	0
C27	0.265077	C19-H22	0.1086	N23-C12-S24	122.64	N3-C4-C6-H11	0	H27	0.30846518	N24-H26	0.1019	H14-C13-H15	108.46	C4-N3-C13-H16	0
H28	0.140068	S25-C26	0.18392	C14-C13-C15	120.02	N3-C12-N23-C13	180	O28	-0.4376531	N24-H27	0.1019	H14-C13-H16	108.46	C4-N7-C5-C6	180
H29	0.140068	S25-C38	0.18392	C14-C13-N23	119.72	N3-C12-S24-C14	0	O29	-0.5569265	C25-O28	0.12183	H15-C13-H16	108.46	C4-N7-C5-C9	180
H30	0.010654	C26-C27	0.15375	C15-C13-N23	119.72	C4-N3-C1-C41	180	H30	0.36444673	C25-O29	0.13513	C19-S18-C31	99.24	C4-S8-C6-C5	0
N31	-0.84604	C26-H28	0.10969	C13-C14-C16	120.02	C4-N3-C12-N23	180	C31	-0.4933691	O29-H30	0.0973	S18-C19-C20	110.27	C4-S8-C6-H17	0
C32	0.616438	C26-H29	0.10969	C13-C14-S24	120.06	C4-N3-C12-S24	180	H32	0.18035552	C31-H32	0.10969	S18-C19-H21	108.76	C5-N7-C4-S8	180
H33	0.346931	C27-H30	0.10969	C16-C14-S24	120.06	C4-C5-C6-H10	0	H33	0.18035552	C31-H33	0.10969	S18-C19-H22	108.76	C6-C5-C9-F10	0
H34	0.337621	C27-N31	0.14647	C13-C15-C17	120.02	C4-C5-C6-H11	0	C34	0.38271984	C31-C34	0.15375	C20-C19-H21	109.56	C6-C5-C9-F11	0
O35	-0.44845	C27-C32	0.15241	C13-C15-H18	119.88	C4-C6-C5-H8	0	H35	-0.0212586	C34-H35	0.10969	C20-C19-H22	109.56	C6-C5-C9-F12	0
O36	-0.54173	N31-H33	0.1019	C17-C15-H18	119.88	C4-C6-C5-H9	0	H36	-0.0212586	C34-H36	0.10969	H21-C19-H22	108.46	C6-S8-C4-N7	0

H37	0.354731	N31-H34	0.1019	C14-C16-C19	120.02	C5-C4-N3-C12	0					C19-C20-H23	109.56	N7-C4-N3-C13	180
C38	-0.16928	C32-O35	0.12183	C14-C16-H20	119.88	C5-C4-C6-H10	0					C19-C20-N24	111.04	N7-C5-C6-S8	180
H39	0.126528	C32-O36	0.13513	C19-C16-H20	119.88	C5-C4-C6-H11	0					C19-C20-C25	111.04	N7-C5-C6-H17	180
H40	0.126528	O36-H37	0.0973	C15-C17-C19	120.02	C5-C6-C4-H7	0					H23-C20-N24	109.88	N7-C5-C9-F10	0
C41	-0.07258	C38-H39	0.10969	C15-C17-H21	119.88	C6-C4-N3-C12	0					H23-C20-C25	108.22	N7-C5-C9-F11	0
H42	0.079329	C38-H40	0.10969	C19-C17-H21	119.88	C6-C4-C5-H8	0					N24-C20-C25	111.14	N7-C5-C9-F12	0
H43	0.079329	C38-C41	0.15375	C16-C19-C17	120.02	C6-C4-C5-H9	0					C20-N24-H26	109.29	S8-C4-N3-C13	180
		C41-H42	0.10969	C16-C19-H22	119.88	C6-C5-C4-H7	0					C20-N24-H27	109.29	S8-C6-C5-C9	180
		C41-H43	0.10969	C17-C19-H22	119.88	H7-C4-N3-C12	0					H26-N24-H27	106.4	C9-C5-C6-H17	180
				C12-N23-C13	104.24	H7-C4-C5-H8	0					C20-C25-O28	123.2	C13-N3-C1-C34	0
				C12-S24-C14	89.47	H7-C4-C5-H9	0					C20-C25-O29	112.73	C13-N3-C1-C34	180
				C26-S25-C38	99.24	H7-C4-C6-H10	0					O28-C25-O29	122.1	S18-C19-C20-H23	0
				S25-C26-C27	110.27	H7-C4-C6-H11	0					C25-O29-H30	106.55	S18-C19-C20-N24	0

				S25-C26-H28	108.76	H8-C5-C6-H10	0					S18-C31-H32	108.76	S18-C19-C20-C25	0
				S25-C26-H29	108.76	H8-C5-C6-H11	0					S18-C31-H33	108.76	S18-C31-C34-H35	0
				C27-C26-H28	109.56	H9-C5-C6-H10	0					S18-C31-C34	110.27	S18-C31-C34-H36	0
				C27-C26-H29	109.56	H9-C5-C6-H11	0					H32-C31-H33	108.46	C19-S18-C31-H32	0
				H28-C26-H29	108.46	C12-N3-C1-C41	180					H32-C31-C34	109.56	C19-S18-C31-H33	0
				C26-C27-H30	109.56	C12-N23-C13-C14	180					H33-C31-C34	109.56	C19-S18-C31-C34	0
				C26-C27-N31	111.04	C12-N23-C13-C15	180					C1-C34-C31	111.04	C19-C20-N24-H26	0
				C26-C27-C32	111.04	C12-S24-C14-C13	180					C1-C34-H35	108.77	C19-C20-N24-H27	0
				H30-C27-N31	109.88	C12-S24-C14-C16	180					C1-C34-H36	108.77	C19-C20-C25-O28	180
				H30-C27-C32	108.22	C13-C14-C16-C19	180					C31-C34-H35	109.8	C19-C20-C25-O29	180
				N31-C27-C32	111.14	C13-C14-C16-H20	180					C31-C34-H36	109.8	C20-C19-S18-C31	0
				C27-N31-H33	109.29	C13-C15-C17-C19	180					H35-C34-H36	107.58	C20-C25-O29-H30	180
				C27-N31-H34	109.29	C13-C15-C17-H21	180							H21-C19-S18-C31	0

				H33-N31-H34	106.4	C13-N23-C12-S24	180							H21-C19-C20-H23	0
				C27-C32-O35	123.2	C14-C13-C15-C17	180							H21-C19-C20-N24	0
				C27-C32-O36	112.73	C14-C13-C15-H18	180							H21-C19-C20-C25	0
				O35-C32-O36	122.1	C14-C16-C19-C17	180							H22-C19-S18-C31	0
				C32-O36-H37	106.55	C14-C16-C19-H22	180							H22-C19-C20-H23	0
				S25-C38-H39	108.76	C14-S24-C12-N23?	0							H22-C19-C20-N24	0
				S25-C38-H40	108.76	C15-C13-C14-C16	180							H22-C19-C20-C25	0
				S25-C38-C41	110.27	C15-C13-C14-S24	180							H23-C20-N24-H26	0
				H39-C38-H40	108.46	C15-C17-C19-C16	180							H23-C20-N24-H27	0
				H39-C38-C41	109.56	C15-C17-C19-H22	180							H23-C20-C25-O28	0
				H40-C38-C41	109.56	C16-C14-C13-N23	180							H23-C20-C25-O28	0
				C1-C41-C38	111.04	C16-C19-C17-H21	180							H23-C20-C25-O28	180
				C1-C41-H42	108.77	C17-C15-C13-N23	180							H23-C20-C25-O29	180

				C1-C41-H43	108.77	C17-C19-C16-H20	180							N24-C20-C25-O28	180
				C38-C41-H42	109.8	H18-C15-C13-N23	180							N24-C20-C25-O29	180
				C38-C41-H43	109.8	H18-C15-C17-C19	180							C25-C20-N24-H26	0
				H42-C41-H43	107.58	H18-C15-C17-H21	180							C25-C20-N24-H27	0
						C19-C16-C14-S24	180							O28-C25-O29-H30	180
						H20-C16-C14-S24	180							O28-C25-O29-H30	0
						H20-C16-C19-H22	180							H32-C31-C34-H35	0
						H21-C17-C19-H22	180							H32-C31-C34-H36	0
						N23-C13-C14-S24	180							H33-C31-C34-H35	0
						S25-C26-C27-H30	0							H33-C31-C34-H36	0
						S25-C26-C27-N31	0								

						S25-C26-C27-C32	0								
						S25-C38-C41-H42	0								
						S25-C38-C41-H43	0								
						C26-S25-C38-H39	0								
						C26-S25-C38-H40	0								
						C26-S25-C38-C41	0								
						C26-C27-N31-H33	0								
						C26-C27-N31-H34	0								
						C26-C27-C32-O35	180								
						C26-C27-C32-O36	180								
						C27-C26-S25-C38	0								
						C27-C32-O36-H37	180								
						H28-C26-S25-C38	0								
						H28-C26-C27-H30	0								

						H28-C26-C27-N31	0								
						H28-C26-C27-C32	0								
						H29-C26-S25-C38	0								
						H29-C26-C27-H30	0								
						H29-C26-C27-N31	0								
						H29-C26-C27-C32	0								
						H30-C27-N31-H33	0								
						H30-C27-N31-H34	0								
						H30-C27-C32-O35	0								
						H30-C27-C32-O35	0								
						H30-C27-C32-O35	180								
						H30-C27-C32-O36	180								
						N31-C27-C32-O35	180								

						N31-C27-C32-O36	180								
						C32-C27-N31-H33	0								
						C32-C27-N31-H34	0								
						O35-C32-O36-H37	180								
						O35-C32-O36-H37	0								
						H39-C38-C41-H42	0								
						H39-C38-C41-H43	0								
						H40-C38-C41-H42	0								
						H40-C38-C41-H43	0								
